# Kyphoplasty with intravertebral reduction devices associated with better height restoration and greater kyphosis correction than kyphoplasty with balloons

**DOI:** 10.1038/s41598-021-84856-9

**Published:** 2021-03-08

**Authors:** Chi-Jung Chiang, Jin-Wei Huang, Shu-Mei Chen, Jiann-Her Lin

**Affiliations:** 1 Department of General Medicine, Taipei Medical University Shuang Ho Hospital, New Taipei City, Taiwan; 2 Division of Neurosurgery, Department of Surgery, School of Medicine, Taipei Medical University, Taipei, Taiwan; 3 Taipei Neuroscience Institute, Taipei Medical University, Taipei, Taiwan; 4 Department of Neurosurgery, Taipei Medical University Hospital, Taipei, Taiwan

**Keywords:** Outcomes research, Chronic pain, Quality of life, Bone

## Abstract

Kyphoplasty (KP) with intravertebral reduction devices (IRD) was reported to be associated with better radiological outcomes than KP with balloons (BK) for osteoporotic vertebral compression fractures (OVCFs). However, the mechanical factors that contribute to the radiological benefits of IRDs require further investigation. To probe the mechanical factors, this retrospective matched cohort study was designed, including the older patients with painful OVCFs and treated with KP. We compared the clinical and radiological outcomes between KP with an IRD and BK, where vertebral body height and kyphotic angle of the cemented vertebrae were measured pre- and postoperatively; clinical outcomes were collected by telephone interviews. The restoration and maintenance ratio suggested that IRDs were associated with favorable effects long-term wise in anterior to middle vertebral body and kyphosis than BK in patients. The gathered results concluded the radiological benefits of IRD regarding both its efficient restoration and maintenance in vertebrae.

## Introduction

Kyphoplasty (KP) is a treatment option for osteoporotic vertebral compression fractures (OVCFs). In contrast to vertebroplasty (VP), vertebral body height expansion and kyphotic angle correction are possible with KP. KP with balloons (BK), which was developed in 1998, employs a combination of an inflatable balloon and bone cement to treat OVCFs with fair outcomes. An instrument with a deflated balloon is inserted into the vertebra and is then inflated to create a space within the fractured body. The balloon is then removed, followed by the injection of bone cement, which hardens relatively quickly, providing strength and stability to the vertebra. Low cement extravasation rates, both short-term and long-term pain relief, and improvements in patient activity levels and quality of life with the use of BK have been reported^1–5^. However, its additional advantages in vertebral height reduction and kyphotic angle reduction have increasingly become controversial following decades of its application. The controversy potentially arises from the overestimated efficacy of BK. Previous studies have revealed significant spontaneous deformity correction of prone position during operation, while the inflatable balloon has not offered significant added benefits^2,6^. In addition, a significant loss of restored height was observed after balloon deflation^2,7^. Subsequently, a controllable intravertebral reduction device (IRD, SpineJack) was designed to provide craniocaudal reduction forces with sustainable height maintenance following the expansion of the device. IRD has been demonstrated to be associated with superior body height restoration and kyphotic angle correction compared with VP^8^. In addition, compared with BK, IRD reportedly resulted in significantly enhanced body height restoration in a human cadaveric study^9^. Another prospective comparative study by Noriega et al. demonstrated the significant radiological benefits of IRD in 15 patients^10^. According to SAKOS study, IRD was noninferior to BK when it comes to OCVF related pain reduction at 12 months from baseline, with significantly greater medial vertebral body height reduction^11^. The purpose of the present duo-center retrospective study was to evaluate the radiological and clinical benefits of IRD over BK with a relatively large sample size and to investigate whether the radiological advantages arise from the restoration effect or the maintenance effect, or both.

## Materials and methods

### Patient selection

The Joint Institutional Review Board at Taipei Medical University Hospital approved the present retrospective duo-center comparative study with a waiver of inform consent (code of approval: N201705068). All experiments were performed in accordance with relevant guidelines and regulations. Patient data was extracted from database of Department of Neurosurgery of Taipei Medical University Hospital and Wan-Fang Hospital within 2013 to 2017. Patients were included if they were older than 60 years; presented with focal back pain and/or lower extremities pain; diagnosed as having OVCFs with an apparent bone edema in the fractured site using MRI T2-weighted short *tau* inversion recovery resonance or using an enhanced area within the vertebral body in MRI-contrast T1-weighted sequences; presented with clinical symptoms corresponding to the locations of the OVCFs. These OVCFs with or without end plate involvement, with intractable pain after conservative treatments, e.g. analgesics, and without signs of neurological impairment in corresponding body parts were indicated to undergo KP in two different modalities (either BK or with IRD) based on the preferences of 9 neurosurgeons in two medical centers. Included patients were expected to exhibit more than 6 months of follow-up radiographs regarding to associated OCVFs locations. Patients were excluded if they were not treated by KP or with any loss of required data.

### Radiological measurements and functional outcomes

**Figure 1:**
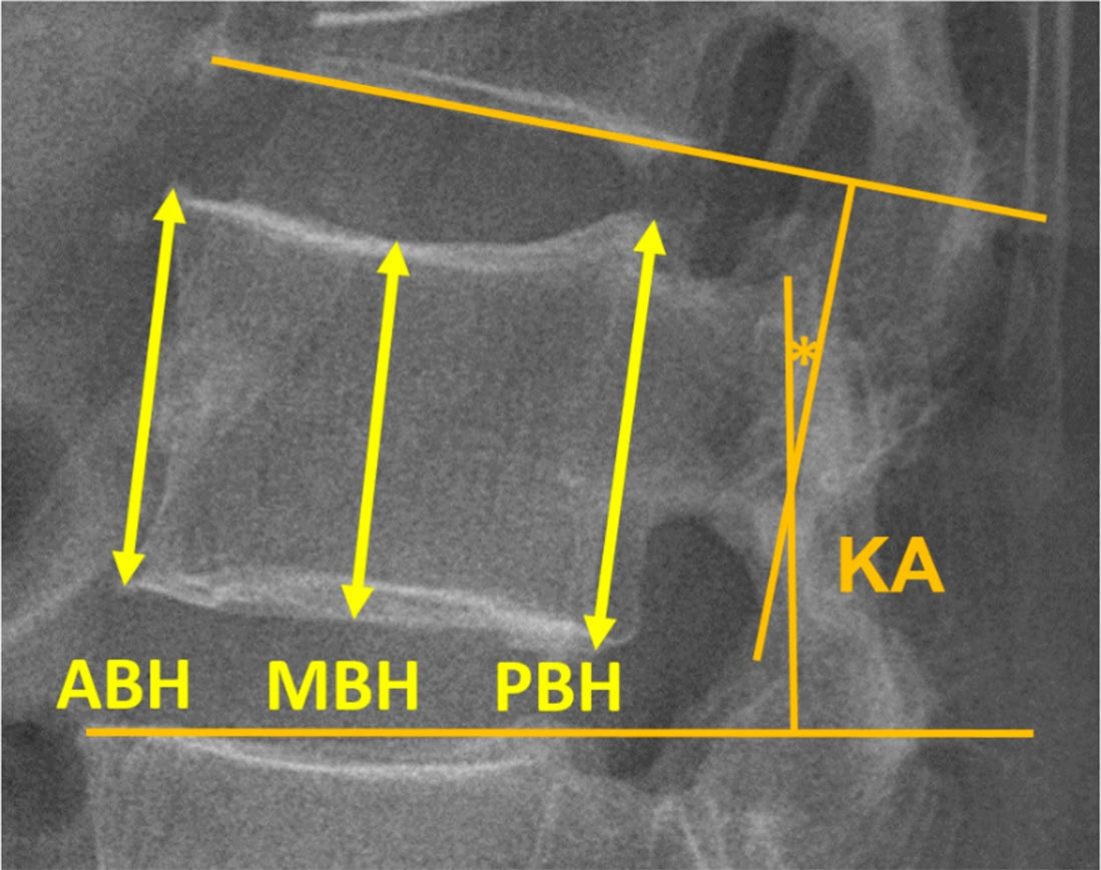
Radiological outcome measurements. (*ABH* anterior body height, *MBH* middle body height, *PBH* posterior body height, *KA* kyphotic angle).

Radiological measurements were obtained from thoracic spine or lumbar spine flexion–extension sagittal radiographs. The preoperative plain films were obtained 1 or 2 weeks prior to operation. The 1-week postoperative and the postoperative final follow-up films, which represented short-term and long-term radiological outcomes, respectively, were obtained. Anterior body height (ABH), middle body height (MBH), posterior body height (PBH), and kyphotic angle (KA) in each of the fractured vertebra were sequentially measured preoperatively (XBH_pre_ and KA_pre_), postoperatively at 1 week (XBH_1w_ and KA_1w_), and at the final follow-up (XBH_f_ and KA_f_). The body heights, including ABH, MBH, and PBH, were defined as the distances between the superior and inferior endplate of the fractured vertebra in the anterior body wall, the center of the body, and the posterior body wall, respectively. The adjacent endplates method was used to assess kyphosis, defining KA based on the intersection of the inferior endplate of the vertebra one level above the superior endplate of the vertebra one level below the fracture (Fig. 1). Radiological parameters were measured by two physicians. Before the study began, both physicians performed radiological assessments on the preoperative radiographs of the same 30 subjects selected randomly from the cohort. Reliability of their measurements was evaluated through intraclass correlation analysis. The reliability of the measurements taken by the two physicians ranged from good to excellent for KA, ABH, MBH, and PBH (Supplementary Table 4).

We used restoration ratio (RR) and maintenance ratio (MR) to compare the short-term restoration and long-term maintenance effects. Height restoration ratio was calculated as follows: Restoration ratio = 1-week postoperative body height − preoperative body height/preoperative body height (XBHRR = XBH_1w_ − XBH_pre_/XBH_pre_). Maintenance ratio was calculated by comparing XBH_1w_ and XBH_f_ (XBHMR = XBH_f_/XBH_1w_). The restoration of KA (RKA) was defined as the difference between KA_pre_ and KA_1w_ (RKA = KA_1w_ − KA_pre_). The maintenance of KA (MKA) was defined as the difference between KA_1w_ and KA_f_ (MKA = KA_f_ − KA_1w_).

Functional outcomes including Visual Analog Scale (VAS) and Oswestry Disability Index (ODI) were obtained through telephonic interviews with the patients or their families.

### Statistical analysis

All statistical analyses were performed using commercially available software (GraphPad Prism 7 Software, Inc., La Jolla, California, USA). Radiological outcomes of different periods in the two groups were compared using two-way analysis of variance. Intergroup comparisons were performed using Student’s *t*-test, results presented as mean ± standard deviation, or Mann–Whitney test, results presented as median (minimum, maximum), for quantitative parameters and chi-square test for the qualitative parameters. A *p* value of < 0.05 denoted a significant difference.

## Results

### Patient characteristics

**Figure 2:**
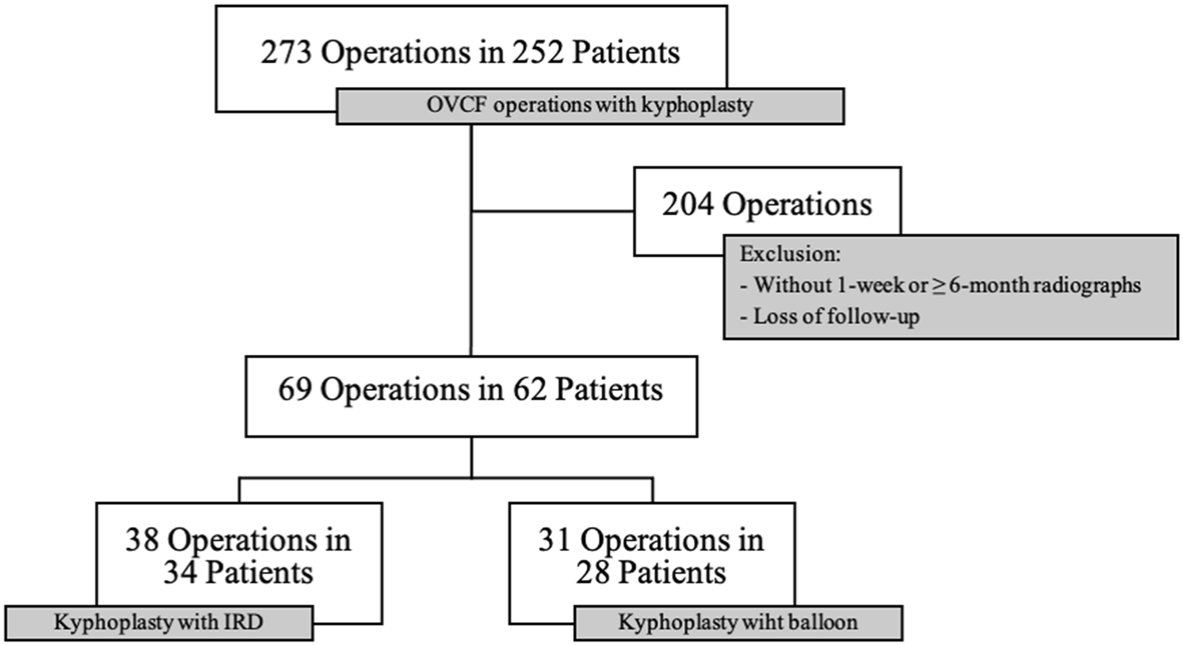
Flowchart illustrating the inclusion process. (*OVCF* osteoporotic vertebral compression fracture, *IRD* intravertebral reduction device).

From year 2013 to 2017, 273 operations in 252 patients over 60 years old were admitted to Neurosurgery department in two hospitals in Taipei, Taiwan for KP treatments for OVCFs. 204 operations were excluded either lacking required follow-up time radiographs or were no longer visiting for further follow-ups. A total of 69 KP operations in 62 patients were included in the present study. The final study cases with required data comprised 28 patients (31 vertebrae) with fractured vertebrae treated with BK and 34 patients (38 vertebrae) treated with IRD. (Fig. 2).

The following demographic characteristics of the included patients were documented: (1) age, (2) sex, (3) bone mineral density (BMD), (4) body mass index (BMI), (5) operation levels, and (6) preoperative radiological parameters. Between patient characteristics, BMD of the IRD group was significantly worse than that of the BK group (*p* = 0.0425). No significant differences within other characteristics were found between the IRD group and the BK group. (Table 1).

**Table 1:** Patient Characteristics and Preoperative radiological data.

	KP with IRD	BK	*p* Value
n	38	31	
Age	72.61 ± 6.68	74.06 ± 6.76	0.373
**Gender**			
F	33	22	0.136
M	5	9	
BMI	23.83 ± 3.12	24.5 ± 4.45	0.488
BMD	0.73 ± 0.14	0.8 ± 0.21	0.163
**OP Level**			
T	13	11	0.912
L	25	20	
**ABH (cm)**			
Preop	1.64 ± 0.49	1.68 ± 0.51	0.789
**MBH (cm)**			
Preop	1.74 ± 0.56	1.77 ± 0.54	0.811
**PBH (cm)**			
Preop	2.66 ± 0.42	2.62 ± 0.41	0.722
**KA (°)**			
Preop	− 4.81 ± 11.93	− 4.78 ± 9.61	0.99

*ABH* anterior body height, *BMD* bone marrow density, *BMI* body mass index, *KA* kyphotic angle, *MBH* middle body height, *PBH* posterior body height, *Preop* preoperative.

### Radiological outcomes

**Figure 3:**
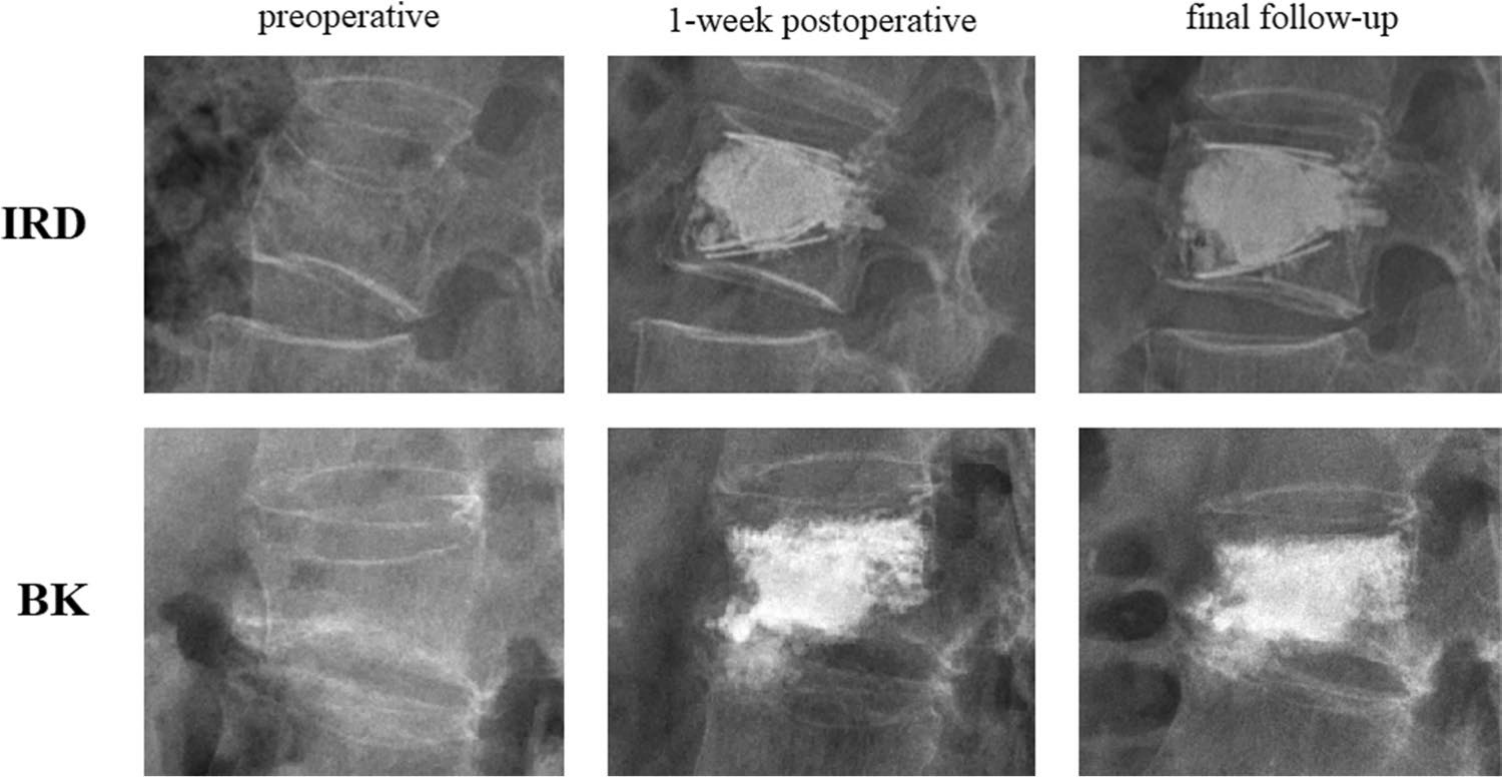
Radiographic demonstration of KP with IRD and BK: preoperative, 1-week postoperative, and final, respectively. (*KP with IRD* kyphoplasty with intravertebral reduction device, *BK* kyphoplasty with balloon).

Among the 69 OVCFs (BK: n = 31; IRD: n = 38), the radiological follow-up rates were both 100% in the short term (1-week post-operation) and in the long term (more than 6 months post-operation). The mean follow-up duration was 11.68 ± 0.99 months in the IRD group and 12.19 ± 0.755 months in the BK group, with a *p* value of 0.6843. No significant differences were observed in the preoperative vertebral body heights and kyphotic angles between the two groups. Two series of vertebral radiographs of both groups were presented in Fig. 3.

**Figure 4:**
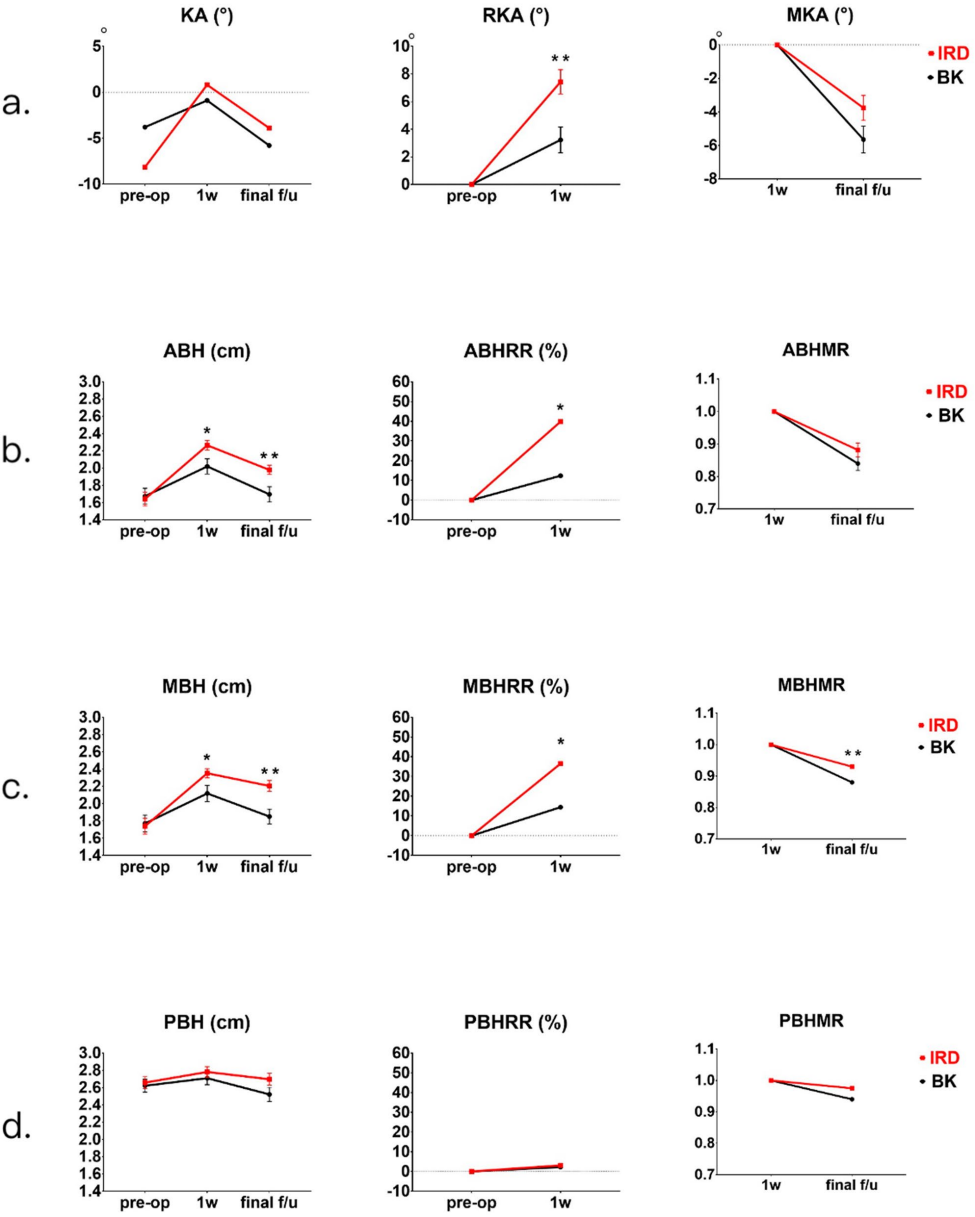
Comparisons of radiological outcomes between the KP with IRD group and the BK group. (**a**) Comparisons of KA at pre-operation, 1-week post-operation, and final follow-up. (**b**) Comparisons of ABH at pre-operation, 1-week post-operation, and final follow-up. (**c**) Comparisons of MBH at pre-operation, 1-week post-operation, and final follow-up. (**d**) Comparisons of PBH at pre-operation, 1-week post-operation, and final follow-up. (*IRD* kyphoplasty with intravertebral reduction device, *BK* kyphoplasty with balloon, *KA* kyphotic angle, *RKA* restoration of kyphotic angle, *MKA* maintenance of kyphotic angle, *ABH* anterior body height, *MBH* middle body height, *PBH* posterior body height, *RR* restoration ratio, *MR* maintenance ratio) (*p* value summary: *p < 0.05, **p ≤ 0.01).

We observed a trend where KA at post-operation was better in the IRD group than in the BK group (*p* = 0.2328 at postoperative 1 week; *p* = 0.0599 at postoperative > 6 months). In addition, KA restorations were significantly more efficient through IRD (IRD vs. BK RKA: 7.425 ± 5.43° vs. 3.23 ± 5.17°, *p* = 0.0017). Although the intergroup difference in KA maintenance was not significant, the mean MKA observed following IRD was better than that following BK (*p* = 0.0896) (Fig. 4a).

Compared with BK, IRD resulted in significantly more favorable short-term and long-term ABHs, with the advantage of a superior restoration effect on ABHs; however, no significant differences were noted between groups when it came to maintenance effect on ABH. (IRD vs. BK: ABH_1w_ 2.26 ± 0.35 cm vs. 2.02 ± 0.49 cm, *p* = 0.0237; ABH_f_ 1.98 ± 0.33 cm vs. 1.695 ± 0.49 cm, *p* = 0.0086; ABHRR *p* = 0.0369; ABHMR *p* = 0.1683) (Fig. 4b). Both the short-term and long-term MBHs were significantly improved in the IRD group compared with the BK group, as well as the restoration and maintenance effects on MBHs (IRD vs. BK: MBH_1w_ 2.35 ± 0.32 cm vs. 2.12 ± 0.52 cm, *p* = 0.0338; MBH_f_ 2.20 ± 0.39 cm vs. 1.85 ± 0.475 cm, *p* = 0.0011; MBHRR *p* = 0.0165; MBHMR *p* = 0.0097) (Fig. 4c). No significant intergroup differences were observed for the posterior part of the vertebral body (Fig. 4d). (Refer to Supplementary Tables 1–3 for detailed statistical outcomes).

### Functional outcomes

Within the 69 OVCFs comprises 34 patients treated with KP with IRD and 28 patients with BK. Out of 34 patients in the IRD group, 16 and 15 patients provided valid VAS and ODI results, respectively. Whereas in the BK group, 10 and 11 out of 28 patients provided valid VAS and ODI results, respectively. Both groups had similar preoperative VAS scores (*p* = 0.0550) and ODI scores (*p* = 0.0513). For each outcome, significant improvements from the baseline were observed in both groups (all *p* < 0.05). After the operation, the VAS score and ODI score in the IRD group significantly decreased from 6.5 to 0 and from 50.44 ± 8.61 to 17.43 ± 5.45, respectively (*p* ≤ 0.005 for both parameters), whereas in the BK group, the scores decreased from 8.5 to 1 and from 74.14 ± 6.57 to 19.19 ± 3.50, respectively (*p* < 0.0001 for both parameters) (Table 2).

**Table 2:** Preoperative and postoperative clinical outcomes.

	KP with IRD	BK	*p* Value
n	16	10	
**VAS**			
Preop	5.63 ± 3.65	8.2 ± 2.44	0.061
Postop	1.75 ± 2.91	1.1 ± 1.1	0.429
	*p* = 0.004**	*p* < 0.0001****	
n	15	11	
**ODI**			
Preop	50.44 ± 33.36	74.14 ± 21.8	0.051
Postop	17.43 ± 21.12	19.19 ± 11.6	0.788
	*p* = 0.002**	*p* < 0.0001****	

*ODI* Oswestry disability index, *Pre-op* preoperative, *Post-op* postoperative.

## Discussion

This present study revealed the radiological benefits of IRD compared to BK for treating OVCFs. In the IRD group, the MBHs at the final follow-up were significantly more favorable, in addition to the restoration and maintenance ratio. The ABHs at the final follow-up also exhibited better improvement and restoration in the IRD group, although there was no significant difference in ABHMR between the two groups. In addition, the KAs at the final follow-up and RKA were significantly more favorable following KP with IRD. The VAS scores and ODI scores also significantly improved compared with the baseline scores in both groups.

Our results demonstrated the restoration of the IRD in the anterior to middle portion of the vertebra based on significantly more favorable ABH and MBH at final follow-up, ABHRR, and MBHRR after KP with an IRD. In addition, the MBHs in the IRD group were maintained at a better level than in the BK group. These parameters indicated superior restoration and maintenance performances of IRD to those of BK especially in the middle portion of the vertebrae. The non-significance of ABHMR could imply that IRD provided less endplate support to the anterior part of the vertebra than the middle part because the wing of IRD could not reach the anterior margin of the vertebral body in most cases. And despite no significance were found in PBHRR and PBHMR of either treatment modalities, it could be explained by the entities of OCVFs rarely exhibited height loss at the posterior portion of the vertebral body. The results of significantly enhanced RKA in the IRD group and the non-significant intergroup differences in MKA suggested that the kyphosis corrections gained from IRD were mainly associated with the restoration effect but not the maintenance effect. The results could be explained by the kyphosis measurement method used in the present study, which involved not only the fractured vertebra but also the superior and inferior vertebral discs that cannot be supported by IRD. The collapse of upper and lower adjacent discs after OVCFs was also a critical factor affecting KA. Several biomechanical studies have reported evidence of the degeneration changes in intervertebral discs and vertebral bodies. A case series study not only observed subsidence of the superior disc but also strong correlations between postoperative vertebral kyphosis and superior disc behavior^12^. The degeneration of intervertebral disc could also lead to stress shielding of the anterior vertebral body by the neural arch, which is associated with a reduction in BMD and inferior cancellous bone architecture anteriorly^13^. The problem of the weakened anterior body particularly arises with flexed vertebrae^14^ and could negatively affect the maintenance of restored body height over time.

Our results revealed the potential radiological advantages of IRD over BK, in addition to the clinical benefits of both procedures. The findings were consistent with those of the SAKOS study, which concluded that IRD had greater potential for body height restoration and maintenance in the long term. In the study, the restorations of MBH obtained with IRD were significantly greater than those obtained with BK at each follow-up period, including 5 days, 1 months, and 12 months^11^. Its previous prospective monocentric comparative study also sustained the similar fashion in restoration of ABH comparing between IRD and BK^10^. In addition, the KA corrections from the baseline of the study were significantly improved in the KP with an IRD group compared with the BK group: − 3.86 vs. − 0.16 at 6 months (*p* = 0.0026) and − 4.44 vs. 0.15 at 12 months (*p* = 0.0012).

The long-term KA corrections in the present study were also significantly improved after KP with an IRD (− 3.66 vs. 2.57, *p* < 0.0001). The wedge angle measurement used for assessing fracture kyphosis in Noriega et al. eliminated the influence of disc degeneration, which could be the reason for the slightly inconsistent results between the two studies. The strengths of our study compared with those of Noriega et al. included a larger study population (38 vs. 16 and 31 vs. 17 fractured vertebrae in the IRD group and the BK group, respectively) enrolled from two medical centers and involving nine neurosurgeons involved in the operations, which potentially resulted in lower sampling bias. In addition, the slightly older patients (mean age of 72.61 ± 6.68 vs. 67.9 ± 4.5 and 74.06 ± 6.76 vs. 68.3 ± 6.1 in the KP with an IRD group and the BK group, respectively) in the present study suggested that IRD promises favorable surgical outcomes for the dominant population with OVCFs.

However, the radiological benefits provided by the use of IRD seemed not to translate into more favorable clinical outcomes. Although our studies revealed significant improvement in VAS and ODI scores from the baseline in both groups, there was no significant difference in each clinical outcome between the IRD and the BK group (VAS score: *p* = 0.4293; ODI score: *p* = 0.7877). Our result coincided with the SAKOS study, which reported progressive improvements in pain intensity or disability, no inclination to either groups was observed when it comes to long term changes (12 months, VAS score: *p* = 0.061; ODI score: *p* = 0.513). However, IRD was reported to provide a more significant reduction in pain intensity than BK in a shorter-term comparison (1 and 6 months)^11^. In addition, the clinical results of the previous prospective monocentric study did not reveal significant differences between the two procedures during the 12-month follow-up period^10^. On the contrary, significantly improved mean ODI scores and EQ-5D scores in the IRD group were observed in the 3-year follow-up results for the same patient cohort, in addition to the greater increase in pain intensity in the BK group^15^, which demonstrated the IRD’s potential to provide long-lasting clinical benefits for OVCF patients. The results also imply that the positive correlation between radiologic advantages and clinical outcome is established only when the follow-up period is adequately long. The duration of patient follow-up rarely exceeded 2 years in previous studies comparing the effectiveness of KP and VP in OVCF treatment, including in comparisons between KP with balloon or IRD and VP. The relative shorter follow-up period of the studies could be the reason for the non-significant clinical advantages of KP^8,16–21^.

In this study, follow-up rates of functional outcomes were low, presumably influenced by the aging population in our study and the relatively long enrollment period. Pain intensity and functional capacity data from relatively small numbers of patients and short follow-up duration limited the capacity of our study to demonstrate the clinical advantages of IRD over BK. The indefinite postoperative period for assessing the functional outcomes could have contributed to the recall bias and influenced the results. The improvement in local kyphotic angle assessed using the adjacent endplate method in the present study could not adequately reveal the sagittal alignment of the spine, which is a potential factor contributing to the overestimation of kyphoplasty in postoperative clinical outcomes. Future multicenter comparative studies with larger study populations and longer follow-up durations could facilitate the clarification of the actual clinical advantages of IRD.

In conclusion, IRD significantly improved ABH, MBH, and KA compared with BK in OVCF patients, and especially benefited MBH in the long term. The advantages of IRD were associated with both its efficient restoration and maintenance of vertebral body heights and kyphotic angle.

## Supplementary Information

Supplementary Tables.
