# Author Correction: Kyphoplasty with intravertebral reduction devices associated with better height restoration and greater kyphosis correction than kyphoplasty with balloons

**DOI:** 10.1038/s41598-025-93093-3

**Published:** 2025-04-01

**Authors:** Chi-Jung Chiang, Jin-Wei Huang, Shu-Mei Chen, Jiann-Her Lin

**Affiliations:** 1 Department of General Medicine, Taipei Medical University Shuang Ho Hospital, New Taipei City, Taiwan; 2 Division of Neurosurgery, Department of Surgery, School of Medicine, College of Medicine, Taipei Medical University, Taipei, Taiwan; 3 Taipei Neuroscience Institute, Taipei Medical University, Taipei, Taiwan; 4 Department of Neurosurgery, Taipei Medical University Hospital, Taipei, Taiwan

Correction to: *Scientific Reports* 10.1038/s41598-021-84856-9, published online 08 March 2021

The original version of this Article contains an error in Affiliation 2, which was incorrectly given as ‘Division of Neurosurgery, Department of Surgery, School of Medicine, Taipei Medical University, Taipei, Taiwan’. The correct affiliation is listed below:

Division of Neurosurgery, Department of Surgery, School of Medicine, College of Medicine, Taipei Medical University, Taipei, Taiwan

