# Supplementary material for: Kyphoplasty with intravertebral reduction devices associated with better height restoration and greater kyphosis correction than kyphoplasty with balloons
Source: Sci Rep. 2021 Mar 8;11:5430. doi: 10.1038/s41598-021-84856-9 (PMC7940421; doi:10.1038/s41598-021-84856-9)
Supplement: Supplementary file 1 — Supplementary Tables. [file 41598_2021_84856_MOESM1_ESM.docx]

**Kyphoplasty with Intravertebral Reduction Devices Associated with Better Height Restoration and Greater Kyphosis Correction than Kyphoplasty with Balloons**

Chi-Jung Chiang^1+^, Jin-Wei Huang^1+^, Shu-Mei Chen^2,3,4^, Jiann-Her Lin^2,3,4^*

1. Department of General Medicine, Taipei Medical University Shuang Ho Hospital, New Taipei City, Taiwan
2. Department of Surgery, School of Medicine, Taipei Medical University, Taipei, Taiwan
3. Taipei Neuroscience Institute, Taipei Medical University, Taipei, Taiwan
4. Department of Neurosurgery, Taipei Medical University Hospital, Taipei, Taiwan

+ Contributed equally

* Corresponding author:

Jiann-Her Lin

Department of Neurosurgery, Taipei Medical University Hospital, Taipei, Taiwan

Division of Neurosurgery, Department of Surgery, School of Medicine, College of Medicine, Taipei Medical University, Taipei, Taiwan

Address: No. 252 Wu-Shing street, Taipei, Taiwan

E-mail: [jiannher@me.com](mailto:jiannher@me.com)

Telephone: +886-970405133

| Supplementary Table 1. Postoperative radiological data | | | |
| --- | --- | --- | --- |
|  | KP with IRD  (n = 38) | BK (n = 31) |  |
| Mean follow-up period (month) | 11.68 ± 0.99 | 12.19 ± 0.755 | p = 0.6843 |
| ABH_1w_ (cm) | 2.26 ± 0.35 | 2.02 ± 0.49 | *p = 0.0237* |
| MBH_1w_ (cm) | 2.35 ± 0.32 | 2.12 ± 0.52 | *p = 0.0338* |
| PBH_1w_ (cm) | 2.78 ± 0.38 | 2.71 ± 0.42 | p = 0.4580 |
| KA_1w_ (°)* | 0.8 (-14.6, 30.8) | -0.9 (-20.5, 14.4) | p = 0.2328 |
| ABH_f_ (cm) | 1.98 ± 0.33 | 1.695 ± 0.49 | *p = 0.0086* |
| MBH_f_ (cm) | 2.20 ± 0.39 | 1.85 ± 0.475 | *p = 0.0011* |
| PBH_f_ (cm) | 2.70 ± 0.42 | 2.52 ± 0.45 | p = 0.1005 |
| KA_f_ (°)* | -3.9 (-25.6, 35.4) | -5.8 (-24, 7.8) | p = 0.0599 |
| - Parameters were analyzed with Student’s *t*-test and presented as mean value ± standard deviation. - Parameters with * are analyzed with Mann-Whitney test, presented as median (minimum, maximum). - Abbreviations: KP with IRD, kyphoplasty with intravertebral reduction device; BK, kyphoplasty with balloon; ABH_1w_, post-operative 1-week anterior body height; MBH_1w_, post-operative 1-week preoperative middle body height; PBH_1w_, post-operative 1-week posterior body height; KA_1w_, post-operative 1-week preoperative kyphotic angle; ABH_f_, anterior body height at final follow-up; MBH_f_, preoperative middle body height at final follow-up; PBH_f_, posterior body height at final follow-up; KA_f_, preoperative kyphotic angle at final follow-up | | | |

| Supplementary Table 2. Restoration of Body heights and Kyphotic angle | | | | |
| --- | --- | --- | --- | --- |
|  | KP with IRD (n = 38) | BK (n = 31) |  |  |
| ABHRR (%)* | 39.77 (-1.255, 180.36) | 12.35 (-25.43, 257.14) | *p = 0.0369* |  |
| MBHRR (%)* | 36.5 (-0.91, 158.90) | 14.42 (-3.68, 140.82) | *p =0.0165* |  |
| PBHRR (%)* | 3.06 (-24.835, 67.43) | 2.21 (-8.0, 16.50) | p = 0.6251 |  |
| RKA (°) | 7.425 ± 5.43 | 3.23 ± 5.17 | *p = 0.0017* |  |
| - Parameters without * are analyzed with Student’s *t*-test, presented as mean ± standard deviation. - Parameters with * are analyzed with Mann-Whitney test, presented as median (minimum, maximum). - Abbreviations: KP with IRD, kyphoplasty with intravertebral reduction device; BK, kyphoplasty with balloon; ABHRR, restoration ratio of anterior body height; MBHRR, restoration ratio of middle body height; PBHRR, restoration ratio of posterior body height; RKA, restoration of kyphotic angle | | | | |

| Supplementary Table 3. Maintenance of Body heights and Kyphotic angle | | | |
| --- | --- | --- | --- |
|  | KP with IRD (n = 38) | BK (n = 31) |  |
| ABHMR | 0.88 ± 0.13 | 0.84 ± 0.12 | p = 0.1683 |
| MBHMR* | 0.93 (0.72, 1.10) | 0.88 (0.62, 1.05) | *p = 0.0097* |
| PBHMR* | 0.975 (0.74, 1.28) | 0.94 (0.75, 1.04) | p = 0.0992 |
| MKA (°) | -3.76 ± 4.59 | -5.65 ± 4.43 | p = 0.0896 |
| - Parameters without * are analyzed with Student’s *t*-test, presented as mean ± standard deviation. - Parameters with * are analyzed with Mann-Whitney test, presented as median (minimum, maximum). - Abbreviations: KP with IRD, kyphoplasty with intravertebral reduction device; BK, kyphoplasty with balloon; ABHMR, maintenance ratio of anterior body height; MBHMR, maintenance ratio of middle body height; PBHMR, maintenance ratio of posterior body height; MKA, maintenance of kyphotic angle | | | |

| Supplementary Table 4. Intraclass correlation analysis | | | | | | | |
| --- | --- | --- | --- | --- | --- | --- | --- |
| Intraclass correlation | | 95% Confidence interval | | *F*-test with true value 0 | | | |
|  |  | Lower bound | Upper bound | Value | df1 | df2 | Sig. |
| ABH (cm) | 0.958 | 0.913 | 0.980 | 46.224 | 29 | 29 | 0.000 |
| MBH (cm) | 0.870 | 0.745 | 0.936 | 14.389 | 29 | 29 | 0.000 |
| PBH (cm) | 0.908 | 0.816 | 0.995 | 20.697 | 29 | 29 | 0.000 |
| KA (°) | 0.990 | 0.979 | 0.995 | 196.661 | 29 | 29 | 0.000 |
| - Abbreviations: ABH, anterior body height; MBH, middle body height; PBH, posterior body height; KA, kyphotic angle; Sig., significance | | | | | | | |

| Supplementary Table 5. Participant characteristics within the BK group | | | | | | | | | | | |  |
| --- | --- | --- | --- | --- | --- | --- | --- | --- | --- | --- | --- | --- |
|  |  | |  | with Functional outcome | | | without Functional outcome | | | | *p*-value | |
| n | | |  | 11 | | |  | 17 | | |  | |
|  | Age | |  | 71.36 | ± | 2.05 |  | 75.82 | ± | 1.52 | 0.0866 | |
|  | Gender | |  |  |  |  |  |  |  |  |  | |
|  |  | *Female* |  | 9 | | |  | 11 | | | 0.3276 | |
|  |  | *Male* |  | 2 | | |  | 6 | | |  |  |
|  | BMI | |  | 25.35 | ± | 1.24 |  | 23.39 | ± | 1.06 | 0.2412 | |
|  | BMD* | |  | 0.82 (0.64, 0.87) | | |  | 0.83 (0.38, 1.235) | | | 0.4887 | |
|  | Level | |  |  | | |  |  | | |  | |
|  |  | *T-spine* |  | 4 | | |  | 7 | | | 0.8424 | |
|  |  | *L-spine* |  | 8 | | |  | 12 | | |  |  |
| - Abbreviation: BK, kyphoplasty with balloon; BMI, body mass index; BMD, bone mineral density - Parameters without * are analyzed with Student’s *t*-test, presented as mean ± standard deviation. - Parameters with * are analyzed with Mann-Whitney test, presented as median (minimum, maximum). | | | | | | | | | | | | |

| Supplementary Table 6. Participant characteristics within the IRD group | | | | | | | | | | | |
| --- | --- | --- | --- | --- | --- | --- | --- | --- | --- | --- | --- |
|  |  | |  | with Functional outcome | | | without Functional outcome | | | | *p*-value |
|  | n | |  | 20 | | |  | 14 | | |  |
|  | Age | |  | 73.55 | ± | 1.53 |  | 71.14 | ± | 1.82 | 0.3200 |
|  | Gender | |  |  |  |  |  |  |  |  |  |
|  |  | *Female* |  | 18 | | |  | 11 | | | 0.3544 |
|  |  | *Male* |  | 2 | | |  | 3 | | |  |
|  | BMI | |  | 23.99 | ± | 0.84 |  | 23.82 | ± | 0.74 | 0.8832 |
|  | BMD* | |  | 0.75 (0.55, 1.25) | | |  | 0.68 (0.57, 0.965) | | | 0.3064 |
|  | Level | |  |  | | |  |  | | |  |
|  |  | *T-spine* |  | 8 | | |  | 5 | | | 0.8814 |
|  |  | *L-spine* |  | 16 | | |  | 9 | | |  |
| - Abbreviation: IRD, intravertebral reduction device; BMI, body mass index; BMD, bone mineral density - Parameters without * are analyzed with Student’s *t*-test, presented as mean ± standard deviation. - Parameters with * are analyzed with Mann-Whitney test, presented as median (minimum, maximum). | | | | | | | | | | | |
